# Use of a highly sensitive two-dimensional luminescence imaging system to monitor endogenous bioluminescence in plant leaves

**DOI:** 10.1186/1471-2229-4-19

**Published:** 2004-11-18

**Authors:** Michel Flor-Henry, Tulene C McCabe, Guy L de Bruxelles, Michael R Roberts

**Affiliations:** 1 Biolumonics Ltd., Staveley Mill Yard, Staveley, nr. Kendal, Cumbria, LA8 9LS, UK; 2 Department of Biological Sciences, Lancaster Environment Centre, Lancaster University, Bailrigg, Lancaster, LA1 4YQ, UK

## Abstract

**Background:**

All living organisms emit spontaneous low-level bioluminescence, which can be increased in response to stress. Methods for imaging this ultra-weak luminescence have previously been limited by the sensitivity of the detection systems used.

**Results:**

We developed a novel configuration of a cooled charge-coupled device (CCD) for 2-dimensional imaging of light emission from biological material. In this study, we imaged photon emission from plant leaves. The equipment allowed short integration times for image acquisition, providing high resolution spatial and temporal information on bioluminescence. We were able to carry out time course imaging of both delayed chlorophyll fluorescence from whole leaves, and of low level wound-induced luminescence that we showed to be localised to sites of tissue damage. We found that wound-induced luminescence was chlorophyll-dependent and was enhanced at higher temperatures.

**Conclusions:**

The data gathered on plant bioluminescence illustrate that the equipment described here represents an improvement in 2-dimensional luminescence imaging technology. Using this system, we identify chlorophyll as the origin of wound-induced luminescence from leaves.

## Background

It is well documented that essentially all living systems spontaneously generate and emit very low levels of light (reviewed in [1]). This ultra-weak bioluminescence is generally characterized by emission of photons (sometimes termed "biophotons") at an intensity less than 10^-14 ^W.cm^-2 ^(< 1000 photons.sec^-1^.cm^-2^). This is in contrast to the more widely known bioluminescence from animals that visibly glow, such as certain species of jellyfish, fireflies and beetles, which employ fluorescent proteins and/or luciferase enzymes to catalyse reactions that result in chemiluminescence. Ultra-weak bioluminescence is generally considered to result from oxidative chemistry occurring within cells [1,2], though in most cases, the source of observed emissions has not been identified. Nevertheless, changes in emission levels in response to stress – particularly oxidative stress – have been observed in many systems, including bacteria [3], plants (*e.g*. [2,4-6]) and animals [7,8]. For this reason, measurement of ultra-weak bioluminescence may be a useful non-invasive technique to monitor rapid perturbations to cellular activity and for early detection of diseased and damaged cells.

In plants, increases in spontaneous low-level luminescence have been observed in response to pathogen infection [5], salt stress [9], osmotic stress [10] and mechanical damage or wounding [2,4,6,11]. It is suggested that luminescence is produced by singlet oxygen and excited carbonyl species generated as a result of lipid peroxidation reactions [2,11]. Lipid peroxidation in wounded and pathogen-infected plant tissues is a common consequence of the generation of ROS, which also act as signals to induce plant defence responses [12].

Quantitative measurements of such ultra-weak photon emissions are normally obtained using sensitive photomultiplier tubes as photon counting devices. However, more recently, 2-dimensional photomultiplier tubes and cooled charge-coupled device (CCD) cameras have also been employed as a means of imaging the spatial distribution of light emission from diseased and damaged plant tissues [2-6,9]. The clearest images were obtained by Chen *et al*., [2], using a micro-channel plate coupled to a cooled CCD to image light captured through a lens. However, the acquisition time required for what is a relatively weak signal, was 1 hour, preventing a detailed temporal investigation of stress-induced bioluminescence.

Here, we present a novel configuration of a cooled CCD that enables high sensitivity, high resolution 2-dimensional imaging with short integration times. We demonstrate the utility of the system to image and quantify delayed chlorophyll fluorescence and wound-induced luminescence from plant leaves.

## Results and Discussion

### Construction of a luminescence imager

**Figure 1:**
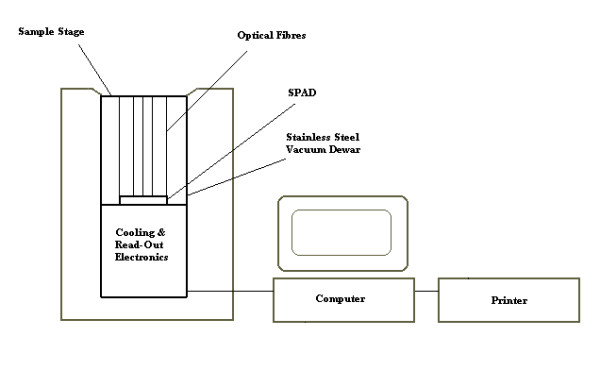
Configuration of the PiXx*ell 1A imager

The experimental imaging set-up, shown in Figure 1, essentially consists of a glass sample stage coupled to a cooled CCD with fibre optics. This configuration enables maximal capture of light emitted from one side of a flat sample placed on the stage by avoiding the inevitable light dispersal associated with lens-based systems. The system is useful for imaging bioluminescence from flat samples such as plant leaves or cells grown transparent culture vessels or on solid supports such as microscope slides or membranes. By analysing the data captured from the CCD, it is possible to estimate the number of photons detected per unit area over a very wide dynamic range. We routinely measured emission levels in the region of 1000 photons/sec/mm^2 ^from wounded leaves (see below). In addition to its high sensitivity, the imager is significantly less expensive than equipment based on photomultiplier tubes.

### Imaging delayed chlorophyll fluorescence

**Figure 2:**
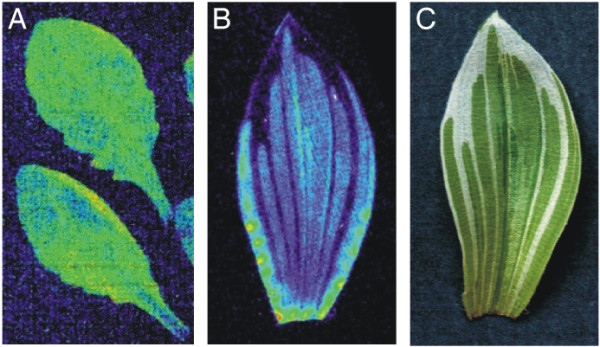
**Delayed chlorophyll fluorescence images. **Luminescence from leaves of *Arabidopsis *(A) and *Tradescantia *(B). Images are 5-minute exposures taken as soon as possible after transfer of leaves to the equipment. A conventional photograph of the *Tradescantia *leaf imaged in (B) is shown to illustrate the pattern of variegation (C).

Delayed chlorophyll fluorescence is a well known phenomenon that is the result of emission of photons from excited chlorophyll molecules following transfer of leaves to the dark. It is generally measured using devices such as photomultiplier tubes and in principle, can be detected by existing lens-based CCD imaging systems. We first tested the spatial and temporal resolution of our system by imaging delayed chlorophyll fluorescence from leaves of *Arabidopsis thaliana *and *Tradescantia albiflora *(Figure 2). The image of a leaf from a variegated *T. albiflora *plant clearly indicates a dependence on chlorophyll for the emission to occur, since white, non-pigmented regions of the leaf do not emit light (compare Figure 2B and 2C). Bright images could be obtained with relatively short exposure times of 1–5 minutes. Delayed chlorophyll fluorescence rapidly subsided between 5 and 10 minutes after transfer to dark. The absolute level of delayed chlorophyll fluorescence in *Arabidopsis *leaves varied greatly, generally appearing to be lower later in the day.

### Spatial and temporal resolution of ultra-weak luminescence in wounded leaves

**Figure 3:**
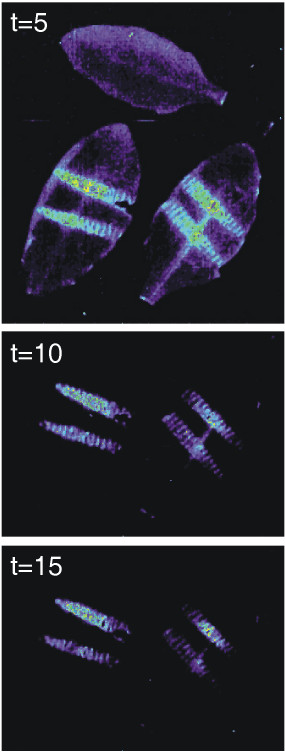
**Wound-induced bioluminescence in Arabidopsis. **Sequential 5-minute exposures of wounded *Arabidopsis *leaves, showing strong luminescence from wound sites. "t = 5" represents an exposure from 0–5 mins after the start of the experiment; "t = 10," 5–10 mins and "t = 15," 10–15 mins. The image from t = 5 min also includes a control, unwounded leaf, which is omitted from subsequent images for brevity.

Delayed chlorophyll fluorescence produces relatively strong light emission compared to typical levels of ultra-weak bioluminescence. To test the sensitivity of our equipment, we wanted to try to image these lower level emissions. Several previous reports had indicated that mechanical wounding increases ultra-weak luminescence from plant leaves, but studies on the temporal and spatial nature of this phenomenon are limited. We used a haemostat to inflict crushing wounds on *Arabidopsis *leaves and imaged them over a time course by making successive 5-minute exposures. In many experiments, no wound-induced luminescence could be seen in the first 5 minutes after transfer to the imager, since it was masked by delayed chlorophyll fluorescence. After this initial period however, photon emission was much more intense around the wounded tissue. In other experiments, where delayed chlorophyll fluorescence was lower, signals could be seen even in the first exposure. Results from such an experiment are shown in Figure 3 and in an accompanying extended animation (Additional File 1). In the first image, which includes an unwounded control leaf and two leaves each wounded twice across the lamina, striking wound-induced luminescence can be seen above a general background of delayed chlorophyll fluorescence (Figure 3A). In the two subsequent 5 min exposures, chlorophyll fluorescence has subsided, whilst the wound-induced luminescence remains high (Figure 3B,3C). The serrated pattern of the haemostat surface is clearly reflected in the pattern of luminescence from the leaf, with areas of greater damage exhibiting maximal photon emission. In general, we observed that induced luminescence is strongest immediately after wounding and then gradually decays over a period of an hour or more.

### Origin of wound-induced luminescence

**Figure 4:**
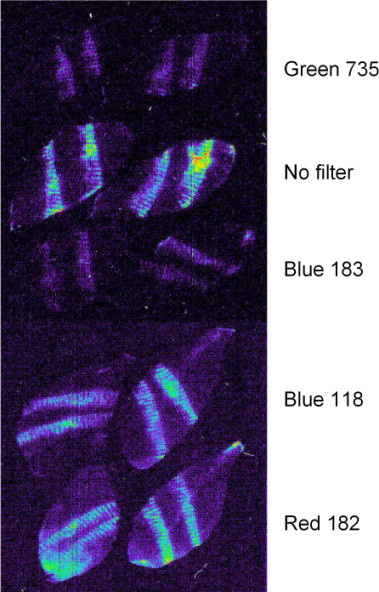
**Spectral characteristics of wound-induced luminescence. **Luminescence images of wounded *Arabidopsis *leaves in the presence of spectral filters. Pairs of wounded leaves are shown imaged with no filter, or with emitted light filtered through green (LEE 735), blue (LEE 183 and LEE 118) or red (LEE 182) filters.

**Figure 5:**
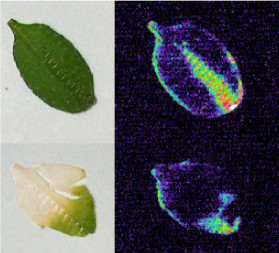
**Chlorophyll is required for wound-induced luminescence. **Luminescence imaging of leaves containing or lacking chlorophyll. The figure shows luminescence images (5 minute exposures) of wounded control (top) and photobleached, norflurazon-treated (bottom), leaves of *Arabidopsis*. Conventional photographs of the leaves imaged are shown at left.

In order to understand more about the origin of the luminescence emitted by wounded leaves, we used coloured filters to investigate its spectral characteristics. The results are shown in Figure 4. A red filter (LEE 182), which transmits visible light of wavelengths greater than 600 nm, was effectively transparent with respect to the light emitted from wound sites, whereas blue and green filters (LEE 183 and LEE 735), which absorb light in the region of 550 – 750 nm, caused a significant reduction in signal reaching the detector when placed between the wounded leaf and the sample stage. Interestingly, a different blue filter (LEE 118), which transmits light above 700 nm, did not significantly affect luminescence (Figure 4). These results suggest that light emitted from wounded *Arabidopsis *leaves has a wavelength between 700 and 750 nm, *i.e*. the red region of the visible spectrum. This is consistent with typical fluorescence from chlorophyll (730 nm). To test whether chlorophyll might be the source of wound-induced photon emission, we used the carotenoid biosynthesis inhibitor, norflurazon, to generate photobleached leaves lacking chlorophyll [13]. Compared with equivalent leaves from control plants, these white leaves failed to luminesce following wounding, except in remaining chlorophyll-containing areas (Figure 5). Together, these results demonstrate that light emission from wounded leaves is chlorophyll-dependent. Since luminescence occurs in the dark, and extends for times well beyond delayed chlorophyll fluorescence, we conclude that excitation energy produced by some wound-induced process is transferred to chlorophyll and emitted as light. One possible source of such energy might be products of oxidative damage, such as excited triplet carbonyls produced during lipid peroxidation [14].

### Temperature-dependence of wound-induced luminescence

**Figure 6:**
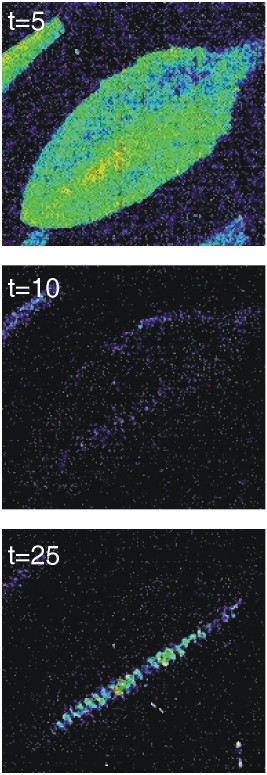
**Temperature-dependence of luminescence. **Images of the same wounded *Arabidopsis *leaf taken at different times following wounding, before and after the application of heat to the sample. "t = 5" represents an exposure from 0–5 mins after the start of the experiment; "t = 10," 5–10 mins and "t = 25," 20–25 mins. The heater in the lid of the sample stage was switched on after 15 mins.

That delayed chlorophyll fluorescence exhibits temperature-dependence has been shown previously in several systems [15]. Since we determined that chlorophyll is the major source of light emission from wounded leaves, we investigated whether wound-induced luminescence might also be affected by temperature. Application of heat to the sample via a foil heater in the lid of the sample chamber significantly increased the intensity of luminescence over the tested temperature range. Without applied heat, the temperature of the sample stage was approximately 7°C, when only low levels of luminescence could be detected. Luminescence increased significantly upon activation of the heater, resulting in an increase in the temperature of the sample stage to something in the region of 20°C. This phenomenon is illustrated in Figure 6, and in an accompanying extended animation (see Additional file 2).

**Figure 7:**
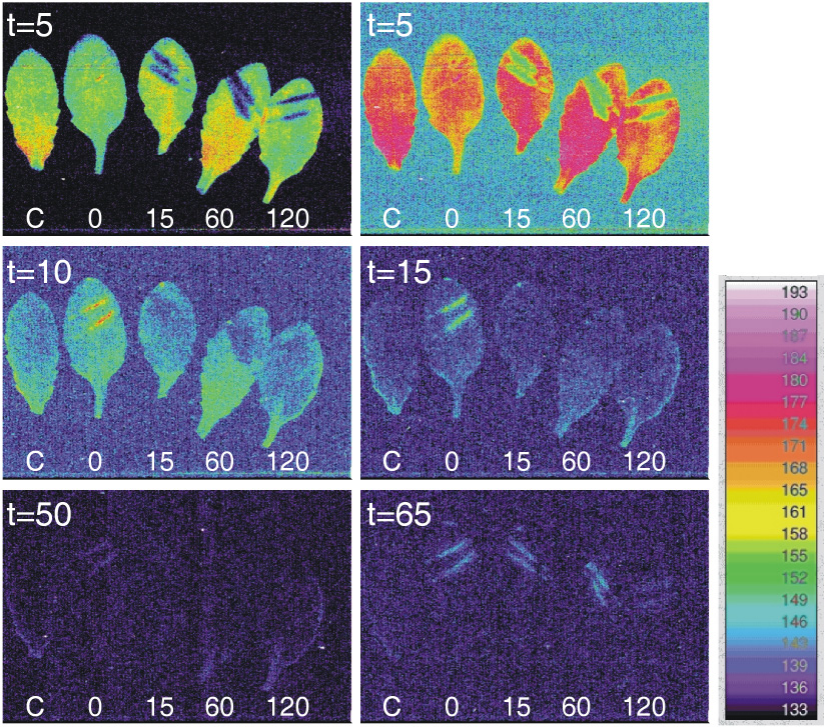
**Heating re-activates luminescence hours after wounding. **5-minute exposures taken at different times during an experiment showing a control *Arabidopsis *leaf (C) and leaves wounded immediately before (0) and 15, 60 and 120 minutes before imaging. The first frame shows the luminescence image using a different display palette from the rest of the images, since it more clearly shows the strong delayed chlorophyll fluorescence emitted by all leaves over the first 5 minutes of the time course, which is absent from the dehydrated areas of the old wounds (but not the freshest wound applied immediately before imaging). All other images are displayed using the same colour palette, which is included in the figure for reference. Times indicated in the upper left corner of each panel represent the time (in minutes) relative to the start of the experiment at which each 5-minute exposure was completed. Heating began at 45 minutes.

Furthermore, we found that heating could activate luminescence in leaves wounded up to 3 hours before imaging. For example, Figure 7 shows a series of images from an experiment in which leaves were wounded at different times before imaging. Without heating, only the leaf wounded immediately before imaging showed detectable wound-induced luminescence, and this emission decayed to very low levels within 20 min. After 45 mins, heating was applied to the sample chamber, and within 10 minutes, wounds applied immediately, 15 mins and 1 hour before the start of imaging were clearly visible, with a faint emission also detectable from a wound applied to the leaf 2 hours before the start of imaging (therefore the image shown here (t = 65) was captured over 3 hours after wounding this particular leaf). This phenomenon is reminiscent of the light emission measured during thermoluminometry [14]. Thermoluminescence is a chlorophyll-dependent light emission observed at high temperatures (maxima at 70–90°C and 120–140°C), which, like ultra-weak bioluminescence, is attributed to singlet oxygen and triplet carbonyls produced by lipid peroxidation during oxidative stress [14]. Coupled with the results above, these data suggest that temperature increases the rate of decay of excited triplet carbonyls, and that the energy released is transferred to chlorophyll and emitted as light. There may also be an additional temperature-dependent component in the rate of emission of light from excited chlorophyll, as observed for delayed fluorescence [15].

## Conclusions

The novel configuration of cooled CCD that we have used in this study to capture 2-dimensional images of plant leaves provides excellent temporal and spatial resolution of bioluminescence/chemiluminescence processes in biological materials, which are important markers of various forms of stress and disease. Using this system, we are able to define the origin of wound-induced luminescence from plant leaves as chlorophyll, and suggest that this arises via the temperature-dependent release of energy from excited triplet carbonyls produced by oxidative stress.

## Methods

### Plant material

Plants of *Arabidopsis thaliana*, Columbia ecotype, were grown in soil in a glasshouse at 22°C with a 16-hour photoperiod. Wounding was performed by crushing leaves with a haemostat. To generate leaves lacking chlorophyll, *Arabidopsis *plants grown for 3 weeks under 16 h light/8 h dark, were watered with 0.1 mM norflurazon (Sigma Aldrich PS1044) and grown under constant illumination at 120 μmol/m^2^/sec. Under these conditions, newly emerging leaf tissue was white. Leaves with white regions were taken 15 days after the start of norflurazon treatment, and used for luminescence imaging.

### Two dimensional luminescence imaging apparatus

The imager used is a two-dimensional imaging system based on a slow-scan 'scientific grade' Silicon Photo Area-Detector [SPAD] (Integrated SPAD Imaging System – PiXx*ell 1A, Biolumonics Ltd), which consists of a cooled ultra-sensitive area photo-detector within a stainless steel vacuum vessel and associated image acquisition electronics. The input image is coupled to the detector with optical fibres (numerical aperture = 1). The detector contains 222,336 pixels, and in the experiments presented here, ran at an operating temperature of -58°C. The combination of the proprietary low read-out noise electronics, cooling of the detector and the optical fibre coupling results in a sensitive imaging system capable of recording the faintest luminescences at high resolution, (40 μm to 200 μm depending on geometry of sample and integration time). The spectral absorption response of the SPAD ranges from the near UV to IR, thus including the entire visible spectrum up to the band-gap energy barrier of Silicon (1.1 eV) *i.e*. at approx 1100 nm; however, for ultimate sensitivity the detector output is monochrome, *i.e*. there is no wavelength selectivity in the present instrument. Further information is available on request from Michel Flor-Henry, Biolumonics Ltd.

### Filters

Coloured filters were obtained from Lee Filters (Andover, UK). We used lighting effect filters LEE 182 – "light red," LEE 118 – "light blue," LEE 183 – "moonlight blue" and LEE 735 – "velvet green." Spectral characteristics of the filters can be viewed at , though readers should note that the transmittance spectra provided with the filters covers the range 300–800 nm, rather than the more limited 400–700 nm spectra shown on the web site. Filters were placed between the leaf and the sample stage prior to imaging.

## Authors' contributions

MF-H designed the PiXx*ell 1A luminescence imager. MF-H and MRR conceived of the current study, participated in its design and coordination, carried out experiments and wrote the manuscript. MF-H performed data analysis. TCM performed the norflurazon experiment, and both TCM and GLdB participated in some experimental design and execution. All authors read and approved the final manuscript.

## Supplementary Material

Additional File 1 Animated gif file showing images from five sequential 5-minute exposures of one control leaf (bottom) and four wounded *Arabidopsis *leaves. Click here for file

Additional File 2 Animated gif file showing images from seven sequential 5-minute exposures of two control leaves (bottom) and four wounded *Arabidopsis *leaves. Images were initially captured from samples on a cold imaging stage, but the heater in the lid of the sample stage was switched on when indicated. Click here for file
