# Supplementary figures and images for: Use of a highly sensitive two-dimensional luminescence imaging system to monitor endogenous bioluminescence in plant leaves
Source: BMC Plant Biol. 2004 Nov 18;4:19. doi: 10.1186/1471-2229-4-19 (PMC535552; doi:10.1186/1471-2229-4-19)

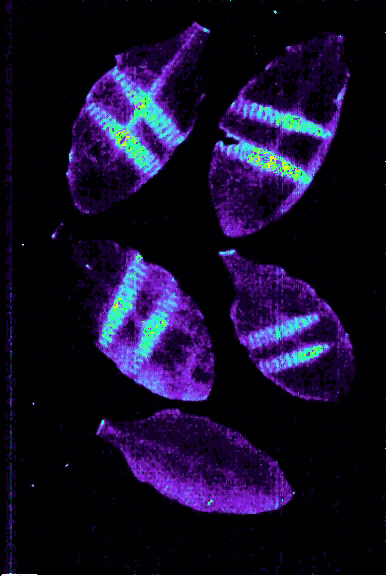

Supplement: Additional File 1 — Animated gif file showing images from five sequential 5-minute exposures of one control leaf (bottom) and four wounded Arabidopsis leaves. [file 1471-2229-4-19-S1.gif]

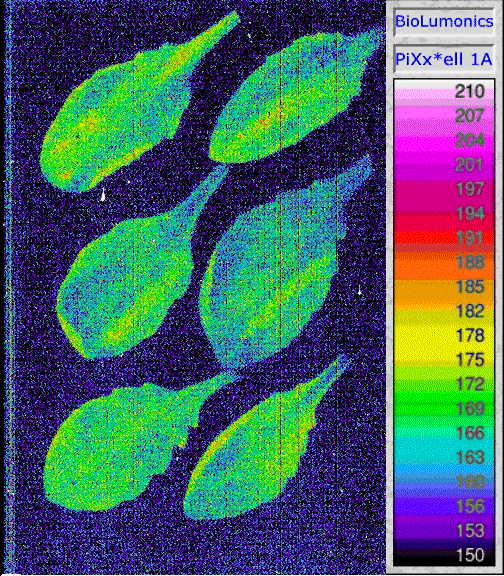

Supplement: Additional File 2 — Animated gif file showing images from seven sequential 5-minute exposures of two control leaves (bottom) and four wounded Arabidopsis leaves. Images were initially captured from samples on a cold imaging stage, but the heater in the lid of the sample stage was switched on when indicated. [file 1471-2229-4-19-S2.gif]
